# Co-infection of HIV and intestinal parasites in rural area of China

**DOI:** 10.1186/1756-3305-5-36

**Published:** 2012-02-13

**Authors:** Li-Guang Tian, Jia-Xu Chen, Tian-Ping Wang, Guo-Jin Cheng, Peter Steinmann, Feng-Feng Wang, Yu-Chun Cai, Xiao-Mei Yin, Jian Guo, Li Zhou, Xiao-Nong Zhou

**Affiliations:** 1 National Institute of Parasitic Diseases, Chinese Center for Disease Control and Prevention, WHO Collaborating Centre for Malaria, Schistosomiasis and Filariasis, Key Laboratory of Parasite & Vector Biology Ministry of Health, Shanghai 200025, China; 2 Anhui Institute of Parasitic Disease Control, Wuhu 241000, China; 3 Fuyang Center for Disease Control and Prevention, Fuyang 236000, China; 4 Department of Epidemiology and Public Health, Swiss Tropical and Public Health Institute, 4051 Basel, Switzerland; 5 University of Basel, 4051 Basel, Switzerland

**Keywords:** HIV, Intestinal parasites, Helminths, Protozoa, Co-infection, China

## Abstract

**Background:**

Intestinal parasite infections (IPIs) are among the most significant causes of illness and disease of socially and economically disadvantaged populations in developing countries, including rural areas of the People's Republic of China. With the spread of the human immunodeficiency virus (HIV) among rural Chinese populations, there is ample scope for co-infections and there have been increasing fears about their effects. However, hardly any relevant epidemiological studies have been carried out in the country. The aim of the present survey was to assess the IPI infection status among a representative sample of HIV-positive Chinese in rural Anhui province, and compare the findings with those from a cohort of non-infected individuals.

**Methods:**

A case control study was carried out in a rural village of Fuyang, Anhui province, China. Stool samples of all participants were examined for the presence of intestinal parasites. Blood examination was performed for the HIV infection detection and anemia test. A questionnaire was administered to all study participants.

**Results:**

A total of 302 HIV positive and 303 HIV negative individuals provided one stool sample for examination. The overall IPI prevalence of intestinal helminth infections among HIV positives was 4.3% (13/302) while it was 5.6% (17/303) among HIV negatives, a non-significant difference. The prevalence of protozoa infections among HIV positives was 23.2% while the rate was 25.8% among HIV negatives. The species-specific prevalences among HIV positives were as follows: 3.6% for hookworm, 0.7% for *Trichuris trichiura*, zero for *Ascaris lumbricoides*, 0.3% for *Clonorchis sinensis*, 1.3% for *Giardia intestinalis*, 16.2% for *Blastocystis hominis*, 1.7% for *Entamoeba *spp. and 8.3% for *Cryptosporidium *spp.. *Cryptosporidium *spp. infections were significantly more prevalent among HIV positives (8.3%) compared to the HIV negative group (3.0%; *P* < 0.05). Among people infected with HIV, *Cryptosporidium *spp. was significantly more prevalent among males (12.6%) than females (4.4%; *P* < 0.05). According to multivariate logistic regression, the factors significantly associated with parasite infections of the people who were HIV positive included sex (male: OR = 6.70, 95% CI: 2.030, 22.114), younger age (less than 42 years old: OR = 4.148, 95% CI: 1.348, 12.761), and poor personal hygiene habits (OR = 0.324, 95% CI: 0.105, 0.994).

**Conclusions:**

HIV positive individuals are more susceptible to co-infections with *Cryptosporidium *spp. than HIV negative people, particularly younger males with poor personal hygiene habits, indicating a need for targeted hygiene promotion, IPI surveillance and treatment.

## Background

Historically, there has been a high prevalence of intestinal parasite infections (IPIs) among human populations in China. Today, IPIs are still common in economically undeveloped rural areas in central China. According to the national survey on important parasitic diseases in the human population completed in 2004, the national prevalence of helminth infections was 21.7%. The prevalence of soil-transmitted helminths (STHs) was 19.6% (hookworms 6.1%, *Ascaris lumbricoides *12.7%, *Trichuris trichiura *4.6%), and the estimated number of individuals infected with STHs was 129 million [1]. With the spread of HIV in China, often in rural areas where transmission was fuelled by illegal blood selling, more and more people living with HIV could be coinfected with parasites [2]. However, hardly any epidemiological studies have explored this issue in China [3].

Recent studies have shown that parasitic infections could disturb the balance of anti-HIV immune responses and contributed to HIV replication [4-6], which could accelerate progress to AIDS [7,8]. The reduced immune response caused by an HIV infection might also lead to a higher susceptibility to parasitic infections. The high prevalence of certain opportunistic parasites among HIV positives is well known [9,10]. Such co-infections present with more severe clinical symptoms compared to parasite infections of otherwise healthy people, and are more difficult to treat [11]. Parasite - HIV co-infections are one of the neglected areas in HIV research although HIV generally has become a major public health concern and research topic in China and beyond. Even since the concerns regarding opportunistic parasite infections among HIV positives have been widely recognized, only few relevant field-epidemiological investigations have been reported in China [3,12].

We carried out a parasitological survey among people living with HIV and non-infected peers in a rural area of Anhui province, China, to understand the epidemiological situation and risk factors for co-infection of HIV and IPIs. The ultimate goal of the study was to provide guidance on the prevention and control of co-infections including treatment needs of HIV/AIDS patients [13], and thus decrease the adverse effects of IPIs on people living with HIV.

## Methods

### Study area and population

The study was conducted in Huangzhuang in the suburbs of Fuyang city, Anhui province, China. In the local clinic for HIV/AIDS treatment a total of 427 HIV-positive people were registered among whom 324 from 12 counties and 126 natural villages were still alive and eligible for inclusion in the study according to the following criteria: age 6-65 years, a signed written informed consent sheet and absence of obvious mental illnesses or other diseases affecting study participation or provision of informed consent. Matching non-HIV infected individuals were recruited among the family members of study participants or, if no suitable controls were available, from their neighborhood. The final study cohort was recruited from 12 villages in Jingjiu district.

### Process of the survey

The study was carried out in the summer of 2008. After a brief public introduction of the study, all residents of the study villages were registered and the participants enrolled in the survey were given a number and a stool collection container with the aim of obtaining a stool sample from each participant. A questionnaire was administered to each participant by fieldworkers from the local Department of AIDS Control and Prevention who had been specifically trained for this task. A blood sample was also collected from all participants and used for HIV testing and hemoglobin, cytokines and CD4+/CD8+ T-lymphocyte determination.

### Laboratory procedures

The blood samples of all participants were screened for anti-HIV antibodies by an enzyme-linked immunosorbent assay (ELISA; Beijing Jinhao Biologic Medicine Company, China). Positive samples were subject to confirmation by Western blot immunoassay (HIV Blot 2.2 WB; Genelabs Diagnostics, Singapore). Tests were conducted in the local Center for Disease Control and Prevention. Hemoglobin was measured using an automatic biochemical analyzer with the diagnostic threshold for anemia set at less than 130 g/L for males and less than 120 g/L for females [14]. CD4+/CD8+ T-lymphocytes were tested using FACSCalibur flow cytometry (BD company, USA). Cytokines quantitative ELISA kits (produced by R & D, U.S.) were used in strict accordance with instructions. The tested cytokines were IL-2, IL-4, IL-10 and IFN-γ. *A. lumbricoides*, hookworm, *T. trichiura *and *Clonorchis sinensis *infections were identified by the Kato-Katz technique [15]. Three Kato-Katz thick smears were prepared from each stool sample. Since hookworm eggs clear very rapidly, the Kato-Katz slides were each read twice, one within 30 mins and one within the hour. *Strongyloides stercoralis *was diagnosed using the Charcoal culture method [16]. *G. intestinalis *and *Entamoeba *spp. were diagnosed by the Lugol's iodine method [1]. *B. hominis *was diagnosed using an *in vitro *culture method [17] and *Cryptosporidium *spp. was detected by modified acid-fast staining [18]. Diagnoses of parasite infections were conducted by staff from the National Institute of Parasitic Diseases, Chinese Center for Disease Control and Prevention together with staff from the Institute of Parasitic Diseases of Anhui province.

### Statistical analysis

EpiData 3.1 was used to establish a database and for double-entry data input by two different individuals. After validation of the database, two identical datasets were obtained, of which one was used for all subsequent analyses. The Student's T-test was employed to test differences in means of age between the HIV positive group and HIV negative group. Univariate statistical analysis was performed using the χ^2 ^test, and the variables with *P* values less than 0.3 in univariate analysis were included in the multivariate model. Multivariate logistic regression modeling was employed to analyze the relationship of socio-demographic, behavioral and immune variables with parasite infection status. All statistical analyses were performed using the SAS 9.1 package.

### Ethical considerations

The study protocol was approved by the institutional review board of the National Institute of Parasitic Diseases, Chinese Center for Disease Control and Prevention in Shanghai. Participants were contacted through the village leaders and the objectives, procedures, and potential risks were carefully explained to all potential participants. Interested individuals provided written informed consent in person or through their parents (in the case of minors) before inclusion in the study. All participants were offered professional counseling before and after HIV testing by staff of the local AIDS prevention and treatment agencies, and all diagnostic results were kept strictly confidential. Free deworming drugs (albendazole, praziquantel) were offered to all participants found to be infected with helminths through local health care institutions.

## Results

### Study cohort

**Figure 1:**
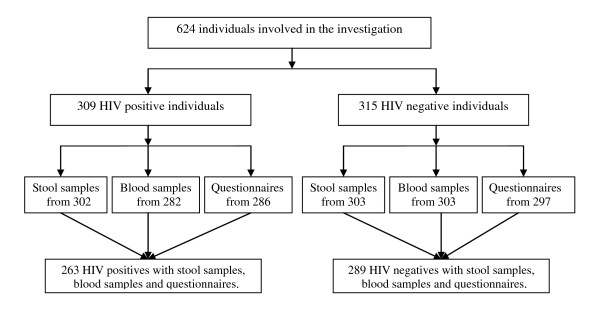
**Participation in a survey on co-infections of HIV and intestinal parasites in a rural area of Fuyang city, Anhui province, People's Republic of China**.

A total of 624 people were recruited, including 309 HIV positives and 315 HIV negative controls. Stool samples were submitted by 605 individuals and blood samples were collected from 585 while questionnaires were answered by 601 participants. Complete data were available from 552 people who had provided stool and blood samples as well as answered the questionnaires (Figure 1).

Among the 605 individuals who had submitted stool samples were 302 HIV positive and 303 HIV negative individuals. The HIV positives comprised 143 (47.4%) males and 159 (52.6%) females and their mean (± SD) age was 42.8 (± 1.2) years. Among the 303 HIV negative individuals, 144 (47.5%) were male and 159 (52.5%) were female and their mean (± SD) age was 41.5 (± 1.3) years. No statistically significant difference in age was found between HIV positives and negatives (T-Tests (Pooled), t = -1.53; *P *> 0.05; Table 1).

**Table 1:** Demographic information of the study participants.

variable	HIV-positive (n = 302) N (%)	HIV-negative (n = 303) N (%)
Sex		
male	143 (47.4%)	144(47.5%)
female	159 (52.6%)	159(52.5%)
Average age* (year)	42.8(41.6, 44.0)	41.5(40.2, 42.7)
Agricultural household*		
yes	258(90.2%)	260(87.5%)
no	28(9.8%)	37(12.5%)
Ethnicity*		
Han	284(99.3%)	293(98.6%)
Others	2(0.7%)	4(1.4%)
Marriage status*		
single	6(2.1%)	16(5.4%)
married	280(97.9%)	281(94.6%)
Educational status*		
illiterate	91(31.8%)	72(24.2%)
primary school	148(51.8%)	100(33.7%)
junior high school	45(15.7%)	110(37.0%)
high school	2(0.7%)	15(5.1%)
college graduates and above	0	0
Occupation*		
student	2(0.7%)	7(2.4%)
farmer	283(98.9%)	286(96.3%)
worker	1(0.4%)	4(1.3%)

* HIV positives n = 286; HIV negatives n = 297

### Parasitic infections

The overall prevalence of intestinal helminth infections was 4.8% (29/605). Hookworm were the most common parasites with 4.0% (24/605), followed by *T. trichiura *and *C. sinensis *(both 0.5%; 3/605). *S. stercoralis *infections were not found (Table 2). People tested HIV positive were infected by hookworm, *T. trichiura *and *C. sinensis *at a rate of 3.6% (11/302), 0.7% (2/302) and 0.3% (1/302) respectively, with no significant difference between infection status with any of these intestinal helminths and HIV serostatus. *A. lumbricoides *infections were not found among HIV positives.

**Table 2:** Parasitic infections of HIV positive and HIV negative study participants

Parasite species	HIV positives (n = 302) % (No. positive)	HIV negatives (n = 303) % (No. positive)	χ^2 ^value	P value
*A. lumbricoides*	0	0.66 (2)		0.4992*
Hookworm	3.64 (11)	4.29 (13)	0.1667	0.6830
*T. trichura*	0.66 (2)	0.33 (1)		0.6238*
*C. sinensis*	0.33 (1)	0.66 (2)		1.0000*
Helminths	4.30 (13)	5.30 (16)	0.3156	0.5742
*B. hominis*	16.23 (49)	22.11 (67)	3.3825	0.0659
*G. intestinalis*	1.32 (4)	0.66 (2)		0.4504*
*Entamoebae*	1.66 (5)	0.99 (3)		0.5045*
*Cryptosporidium *spp.	8.28 (25)	2.97 (9)	8.0399	0.0046
Protozoa	23.2 (70)	25.80 (76)	0.2994	0.5843

* tested by Fisher exact test, others tested by chi-square test

The overall prevalence of intestinal protozoa was 24.1% (146/605). *B. hominis *was diagnosed most often (19.2%; 116/605), followed by *Cryptosporidium *spp. which was found in 5.6% (34/605) of all study participants. HIV positives were infected with *B. hominis, Cryptosporidium *spp., *G. intestinalis *and *Entamoebae *spp. at a rate of 16.2% (49/302), 8.3% (25/302), 1.3% (4/302) and 1.7% (5/302) respectively. A significant difference between rates among HIV positives and negatives was only found in the case of *Cryptosporidium *spp. infections, which were more common among HIV positives (*P*< 0.05; Table 2). Significantly more of these *Cryptosporidium *spp. infected HIV positives were males (prevalence: 12.6%) compared with females (prevalence 4.4%; *P *= 0.010).

### Multiparasitism

The prevalence of intestinal parasite infections among the HIV positives was 26.2%. Most common were single species infections (66 out of 79 or 83.5% of the parasite-infected HIV positive individuals) while 9 (11.4%) were infected with two species concurrently and 3 (3.8%) with three species. One individual was infected with four intestinal parasite species concurrently.

### Risk factors for coinfection with HIV and *Cryptosporidium *spp. or *B. hominis*

Among 263 HIV positives that had answered the questionnaire, *Cryptosporidium *spp. prevalence was significantly higher among males than females (OR = 6.70, 95% CI: 2.03 - 22.11) and those younger than 42 years (OR = 4.15, 95%CI: 1.35 - 12.76). Individuals were at lower risk if they had IL-2 less than 77 pg/ml (OR = 0.23, 95%CI: 0.08 -0.67) or good hygiene habits (OR = 0.32, 95%CI: 0.11 - 0.99; Table 3). There was also a significant difference in the prevalence of *B. hominis *between females (21.9%) and males (11.9%, p < 0.05) in people who lived with an HIV positive person. The multivariate logistic regression analysis showed that nutritional status was significantly associated with *B. hominis *infection (OR = 0.26, 95% CI: 0.07 - 0.95; Table 4).

**Table 3:** Multivariate logistic regression analysis of risk factors for HIV and *Cryptosporidium *spp.coinfection.

Variables	Regression coefficient	Standard Error	OR value (95%CI)	χ^2 ^value	*P *value
male(1 = yes, 0 = no)	1.9022	0.6092	6.700 (2.030, 22.114)	9.7496	0.0018
age < 42 years (1 = yes, 0 = no)	1.4225	0.5734	4.148 (1.348, 12.761)	6.1551	0.0131
IL-2 < 77(pg/ml) (1 = yes, 0 = no)	-1.4872	0.5573	0.226 (0.076, 0.674)	7.1208	0.0076
Good habits (1 = yes, 0 = no)	-1.1275	0.5724	0.324 (0.105, 0.994)	3.8803	0.0489

**Table 4:** Multivariate logistic regression analysis of risk factors for HIV and *B.hominis *coinfection

Variables	Regression coefficient	Standard Error	OR value (95%CI)	χ^2 ^value	P value
Male (1 = yes, 0 = no)	-0.6713	0.3643	0.511 (0.250, 1.044)	3.3967	0.0653
IL-2 < 77(pg/ml) (1 = yes, 0 = no)	0.4622	0.3587	0.630 (0.312, 1.272)	1.6609	0.1975
Good nutrition (1 = yes, 0 = no)	-1.3350	0.6525	0.263 (0.073, 0.945)	4.1861	0.0408
Good habit (1 = yes, 0 = no)	-0.6422	0.3824	0.526 (0.249, 1.113)	2.8202	0.0931

## Discussion

Among the 605 individuals included in the present study, the prevalence of intestinal helminths was 4.8%, with hookworm being the most common species, followed by *T. trichiura *and *C. sinensis*. The prevalence of *A. lumbricoides *was 0.3%. These values are considerably lower than those reported from the nationwide survey of important human parasites in China conducted from 2001 to 2004 where the prevalence of hookworm was 6.1% and that of *A. lumbricoides *12.7% [1]. The economical development of the country resulting in increased urbanization, infrastructure development and increased health consciousness [19] including a reduction of nightsoil use as well as a relatively old study population (average age 42 years) probably all contributed to this apparent decline.

It has been argued that a HIV infection would increase the risk of intestinal helminth infection [20,21], but the results of the present study do not support this claim, consistent with results reported by Nielsen *et al. *[22,23]. One reason might be that HIV positive individuals change their health-related behavior more radically than their HIV negative peers as they received much more health care attention following the HIV diagnosis. The measured prevalence of *B. hominis *was 19.2%, higher than other values reported from China [24-28] but consistent with the findings from other studies where a similar diagnostic approach was followed [29,30]. In contrast to reports that HIV positives were more susceptible to *B. hominis *[31,32], no significant difference in *B. hominis *prevalences between HIV positives and HIV negatives was found in the present study. Interestingly, the *B. hominis *prevalence among females was significantly higher than among males in the HIV positive group but no such difference was observed in the HIV negative population.

The only parasite that was found significantly more often among HIV positives than among HIV negatives was *Cryptosporidium *spp., confirming the findings of numerous other studies [33-35]. According to the multivariate logistic regression analysis, males younger than 42 years and with poor hygiene habits were particularly at risk of *Cryptosporidium *spp. infection. This mirrors findings by Hunter *et al. *[36]. Thus, this population should receive particular attention with regard to hygiene education and targeted anti-parasitic treatment.

In this present study, we find that the people with IL-2 ≥ 77(pg/ml) were more susceptible to coinfection with HIV and *Cryptosporidium *spp., which indicate that T lymphocytes are involved in the immune response to the co-infection, although a decrease of IL-2 was observed with the HIV infection. The mechanism needs further study in the future, since IL-2 has a key role in T lymphocyte proliferation and activity and is fundamental to a human protective immune response [37].

A number of *Cryptosporidium *species infect humans, namely *C. parvum, C. hominis, C. muri *and *C. meleagridis *[38-42]. More work is needed to identify the particular *Cryptosporidium *species and genotypes prevalent in China.

## Conclusions

HIV positive individuals are more susceptible to co-infections with *Cryptosporidium *spp. than HIV negative people, particularly younger males with poor personal hygiene habits, indicating a need for targeted hygiene promotion, IPIs surveillance and treatment.

## Competing interests

The authors declare that they have no competing interests.

## Authors' contributions

Conceived and designed the experiments: L-GT X-NZ J-XC T-PW. Performed the experiments: L-GT F-FW JG X-MY W-DW L-HL. Analyzed the data: L-GT. Contributed reagents/materials/analysis tools: G-JC Y-CC LZ. Wrote the paper: L-GT PS X-NZ. All authors read and approved the final version of the manuscript.
